# Difficult Cases

**Published:** 1886-10-02

**Authors:** 


### Difficult Cases.

*The intention of this column is to afford assistance to clergymen, district visitors, hospital officials, medical men, and others who have to provide for the treatment or accommodation of difficult cases of disease, sometimes in its acute, but usually in its chronic form. Anyone being in doubt as to the best course to pursue in any such case, is invited to communicate with the Editor. All such communications should be written on one side of the paper only, and be addressed to the Editor, the envelope being marked in the top left-hand corner, "Difficult Cases."*

### An Intractable Child.

F. F. G. writes : "I shall be greatly obliged if any of your readers would kindly inform me of any school to which a child nine years old could be sent. She is one of four or five children, none of whom can speak intelligibly—saying only a few simple words, and those only half-articulated. She has had no illness, but is very perverse, and develops frequently a strong tendency to mischief and destruction, tearing clothing, breaking windows and furniture, etc. Such fits last about a day ; in the intervals she is very disobedient and intractable.

"I regard them as evidences of epileptiform seizures, with tendency to insanity. The parents are related (cousins, I believe) ; no other family history. They are able to pay."

*** The case referred to seems to be one suitable for admission to an idiot asylum as a paying patient, where the child will be subject to discipline, and where it will receive a sound education, combined with adequate care, nursing, and medical treatment. A letter should be addressed to the Medical Superintendent, The Asylum for Idiots, Earlswood, Surrey, or to the Royal Albert Asylum, Lancaster, where such cases are received on payment of sums varying from £63 to £210 per annum inclusive. Or "F. F. G." might write to Mr. A. Vard, Bearsted House Asylum and School, Maidstone.

